# Supplementary material for: Stretching of the retinal pigment epithelium contributes to zebrafish optic cup morphogenesis
Source: eLife. 2021 Sep 21;10:e63396. doi: 10.7554/eLife.63396 (PMC8530511; doi:10.7554/eLife.63396)
Supplement: Supplementary file 1. [file elife-63396-supp1.docx]

**Table S1. List of primers used in this study**

| **Amplicon** | **Forward** 5’ to 3’ | **Reverse** 5’ to 3’ |
| --- | --- | --- |
| *bhlhe40* Promotor | G AATAGGCTGTCCATGTGGTC | CAAGCCTCAGAAGTAGGACG |
| *bhlhe40* E1 | GTGTAAGGGATGGTCAACAGTG | CAGTTGGGTCAGTTTGAGTTCG |
| *bhlhe40* E2 | GCTTGATGTGTGGACGTTAC | TGTCGCATCACCAGGCTATC |
| *bhlhe40* E3 | GTCCTTGCATGTCAGTGTTTAG | GTAAATCAGCGTTCATCCCAC |
| *bhlhe40*E4 | ACACTGTACGCTTATGGGAG | CCAGAACACCAGGGATAGAC |
| *STMN1* | GGAA**AGGCCT**ATGGCTTCTTCTGATATCCAGGTG | GGAA**AGGCCT**TTAGTCAGCTTCAGTCTCGTC |
| *bhlhe40* probe | TGCTACGTAAAAGAAAGCGGG | *TCCATTAACCCTCACTAAAGGGAA*TTCGGGAGCTTATTCAGCAGG |

StuI restriction site introduced for cloning purposes is highlighted in bold. The sequence of the T3 promoter for probe synthesis is highlighted in italic
