# Supplementary material for: Stretching of the retinal pigment epithelium contributes to zebrafish optic cup morphogenesis
Source: eLife. 2021 Sep 21;10:e63396. doi: 10.7554/eLife.63396 (PMC8530511; doi:10.7554/eLife.63396)
Supplement: Transparent reporting form [file elife-63396-transrepform1.docx]

***eLife’s* transparent reporting form**

We encourage authors to provide detailed information *within their submission* to facilitate the interpretation and replication of experiments. Authors can upload supporting documentation to indicate the use of appropriate reporting guidelines for health-related research (see [EQUATOR Network](http://www.equator-network.org/%20)), life science research (see the [BioSharing Information Resource](https://biosharing.org/)), or the [ARRIVE guidelines](http://www.plosbiology.org/article/info:doi/10.1371/journal.pbio.1000412) for reporting work involving animal research. Where applicable, authors should refer to any relevant reporting standards documents in this form.

If you have any questions, please consult our Journal Policies and/or contact us: [editorial@elifesciences.org](mailto:editorial@elifesciences.org).

**Sample-size estimation**

- You should state whether an appropriate sample size was computed when the study was being designed
- You should state the statistical method of sample size computation and any required assumptions
- If no explicit power analysis was used, you should describe how you decided what sample (replicate) size (number) to use

Please outline where this information can be found within the submission (e.g., sections or figure legends), or explain why this information doesn’t apply to your submission:

The number of zebrafish embryos or number of cells analyzed in each experiment was determined on the basis of previous published studies with similar analysis and studies (see for example Sidhaye J, Norden C. Concerted action of neuroepithelial basal shrinkage and active epithelial migration ensures efficient optic cup morphogenesis. Elife. 2017 Apr 4;6; Nicolas-Perez M, Kuchling F, Letelier J, Polvillo R, Wittbrodt J, Martinez-Morales JR. Analysis of cellular behavior and cytoskeletal dynamics reveal a constriction mechanism driving optic cup morphogenesis. Elife. 2016 Oct 31;5). The sized ranged between 15 and 20 embryos.

A total of 50 wild type embryos were injected for the generation of Tg(E1-*bhlhe40*:GFP) zebrafish line. According to previous experiences in the lab while generating zebrafish transgenic lines, this number was considered sufficient to obtain at least 3-4 founders. Some embryos die of natural causes during development, others because of the injection and most embryos show somatic integration of the transgene. Indeed, as expected, within the whole bunch we were able to identify 3 fishes with germinal integration and no further injection was needed.

Movies and 3D reconstructions reported in this study to show the reporter pattern were performed in one representative embryo as it is usually done in zebrafish studies.

Kaede photoconversion experiments were performed in 19 embryos. Initially 20 embryos were injected with Kaede mRNA. Some embryos died of natural cause, other after irradiation and a few showed photoconverted cells outside the RPE. A total of 12 embryos showed the expected pattern of RPE and CMZ cells photoconverted. This experiment was based in previous information regarding fate maps from other authors and in the reporter’s expression pattern, therefore we considered 12 embryos sufficient to validate RPE cells origin.

This information relates to figure 1.

Apical surface was measured in 15-20 cells. The cells appeared rather homogeneous and the difference very significant with the used size. We concluded that no additional sampling was needed.

This information relates to Figure 2.

Figure 3 – Data reported in Fig 3 were determined using the movies shown in this study

In Blebbistatin, Azidoblebbistatin and Nocodazole treatments we used between 15 and 40 embryos. In those cases, in which the experiment was completely new we increased the sample size to 40 in order to get more information.

This information relates to Figure 2.

This information relates to Figures 5 and 6.

Data reported in figure 5 and 6 were obtained from 7 embryos analyzed in one experiment. Experiments were performed 3 times obtaining similar results.

This information relates to Figures 7

Data reported were obtained from the analysis of 3-20 embryos form medaka, mouse and chick. Only 1 human embryos per stage was analyzed.

**Replicates**

- You should report how often each experiment was performed
- You should include a definition of biological versus technical replication
- The data obtained should be provided and sufficient information should be provided to indicate the number of independent biological and/or technical replicates
- If you encountered any outliers, you should describe how these were handled
- Criteria for exclusion/inclusion of data should be clearly stated
- High-throughput sequence data should be uploaded before submission, with a private link for reviewers provided (these are available from both GEO and ArrayExpress)

Please outline where this information can be found within the submission (e.g., sections or figure legends), or explain why this information doesn’t apply to your submission:

Each experiment was repeated from a minimum of 3 to a maximum of 6 times.

We did not exclude samples unless embryos died in the course of the experiments as stated above,

No outliers were found

**Statistical reporting**

- Statistical analysis methods should be described and justified
- Raw data should be presented in figures whenever informative to do so (typically when N per group is less than 10)
- For each experiment, you should identify the statistical tests used, exact values of N, definitions of center, methods of multiple test correction, and dispersion and precision measures (e.g., mean, median, SD, SEM, confidence intervals; and, for the major substantive results, a measure of effect size (e.g., Pearson's r, Cohen's d)
- Report exact p-values wherever possible alongside the summary statistics and 95% confidence intervals. These should be reported for all key questions and not only when the p-value is less than 0.05.

Please outline where this information can be found within the submission (e.g., sections or figure legends), or explain why this information doesn’t apply to your submission:

Figure 2F.

Figure 3 -

Figure 4K

U Mann-Whitney test (SPSS)

Figure 4L

U Mann-Whitney test (SPSS)

Figure 4M

U Mann-Whitney test (SPSS)

Figure 4N

U Mann-Whitney test (SPSS)

Figure 5I

U Mann-Whitney test (SPSS)

Figure 5J

U Mann-Whitney test (SPSS)

Figure 6B

Kruskal-Wallis test

U Mann-Whitney test (SPSS) between 17hpf y 20 hpf

(For large datasets, or papers with a very large number of statistical tests, you may upload a single table file with tests, Ns, etc., with reference to sections in the manuscript.)

**Group allocation**

- Indicate how samples were allocated into experimental groups (in the case of clinical studies, please specify allocation to treatment method); if randomization was used, please also state if restricted randomization was applied
- Indicate if masking was used during group allocation, data collection and/or data analysis

Please outline where this information can be found within the submission (e.g., sections or figure legends), or explain why this information doesn’t apply to your submission:

**Additional data files (“source data”)**

- We encourage you to upload relevant additional data files, such as numerical data that are represented as a graph in a figure, or as a summary table
- Where provided, these should be in the most useful format, and they can be uploaded as “Source data” files linked to a main figure or table
- Include model definition files including the full list of parameters used
- Include code used for data analysis (e.g., R, MatLab)
- Avoid stating that data files are “available upon request”

Please indicate the figures or tables for which source data files have been provided:

Source data files have been provided for data presented in

Figure 2, Figure 3, Figure 3-figure supplement 1, Figure 4, Figure 5, Figure 6, Figure 7.
